# Continuity of Chronic Disease Care in Gaza: a Commentary on Health System Collapse and Humanitarian Imperatives

**DOI:** 10.1007/s44197-025-00489-5

**Published:** 2025-11-21

**Authors:** Mohd Sharique Katchhi, Long Chiau Ming

**Affiliations:** 1 Department of Pharmacy Practice, KLE College of Pharmacy, Bengaluru, 560010 India; 2 KLE Academy of Higher Education and Research, Belagavi, Karnataka 590010 India; 3 Sir Jeffrey Cheah Sunway Medical School, Faculty of Medical and Life Sciences, Sunway University, Sunway City, Malaysia; 4 Datta Meghe College of Pharmacy, Datta Meghe Institute of Higher Education and Research (deemed to be a University), Sawangi (M), Wardha, India

**Keywords:** Non-communicable diseases (NCDs), Continuity of care, Health systems resilience, Humanitarian crisis, Gaza conflict

## Abstract

The 2023 Gaza conflict precipitated a collapse of essential health services, exposing the extreme fragility of chronic disease care in humanitarian crises. Non-communicable diseases (NCDs) account for more than 70% of deaths in Palestine, yet the management of hypertension, diabetes, cardiovascular disorders, and renal failure has been critically disrupted by the destruction of infrastructure, blockade of medical supplies, and mass displacement. This work synthesizes recent peer-reviewed literature, WHO and UN reports, and field assessments from 2023 to 2025 examining the continuity of NCD care in Gaza. Adherence to medical follow-up among NCD patients has declined from 96.7% before the war to 40.7% amid ongoing conflict, while more than 90% of primary healthcare facilities report shortages in insulin, antihypertensives, and dialysis supplies. Only 14 of 36 hospitals remain partially functional, leaving thousands without life-sustaining treatment. The findings reveal how conflict transforms chronic, manageable diseases into acute emergencies. Sustaining NCD care must therefore be recognized as a humanitarian and ethical imperative, requiring an immediate ceasefire and restoration of essential health system functions.

## Introduction

When the war in Gaza erupted in October 2023, it shattered not only homes and hospitals but the fragile threads that held everyday life together [1, 2]. In 2023, non-communicable diseases (NCDs) accounted for nearly 71.8% of all deaths in Palestine with hypertension, diabetes, and cardiovascular disorders leading the burden [3]. The specific burden within Gaza is now better characterized by recent survey data, the self-reported prevalence of common NCDs, and risk factors includes 25.6% of adults reporting diabetes, 40.4% reporting hypertension, 21.9% reporting dyslipidemia, and 9.8% reporting asthma or COPD [4]. In the 2020 survey, 49.3% of adults with both hypertension and type 2 diabetes lived in large households, 53.8% had low physical activity, and passive or never-smokers predominated, while 62.3% were female, illustrating the clustering of demographic and lifestyle risk factors that exacerbate chronic disease in Gaza [5, 6].

Amid the sounds of airstrikes and sirens, thousands of people living with chronic illnesses – those dependent on daily insulin injections, blood pressure medicines, or dialysis sessions – found their lifelines abruptly severed [1, 7]. For them, survival does not depend on emergency surgery or battlefield triage, but something far quieter: the simple continuity of care [7]. Yet, in the chaos of conflict, this continuity has become almost impossible. Hospitals that once managed diabetes clinics now lie in ruins. Pharmacy shelves are empty [8]. Reports indicate that Ministry of Health Primary Care Centers (MPs) experience severe shortages in essential medications, with insulin, antiepileptics, and cancer treatments unavailable in over 90% of cases, and critical deficiencies in antibiotics and psychiatric medication. Equipment shortages, including limited oxygen supplies and diagnostic tools, further strain trauma and chronic disease care. Only 39% of MPs provide maternity and vaccination services, while mental health services are nearly absent [8]. Patients walk for miles carrying their medical records in plastic bags, searching for functioning facilities or humanitarian convoys. While the world’s attention focuses on trauma and acute injuries, those living with chronic conditions are silently deteriorating, caught in a health emergency that unfolds slowly, invisibly, and often without witness. The war has transformed manageable diseases into life-threatening crises, exposing how fragile healthcare becomes when stability disappears. Ensuring the continuity of chronic disease care in Gaza is not merely a medical obligation, but an act of preserving dignity, humanity, and the right to live beyond survival [9].

## The Cascading Health Crisis: from Chronic To Acute

The interruption of care for NCDs in Gaza has ignited a cascading health crisis, rapidly transforming manageable chronic conditions into acute, life-threatening emergencies. For thousands of Palestinians, the daily battle for survival is not against explosives, but against the silent deterioration of their own bodies. The consequences of this breakdown in care are both predictable and devastating, supported by a growing body of evidence from this and other conflicts.

A recent study conducted during the conflict paints a stark picture of this reality. Adherence to regular medical follow-ups among patients with NCDs has plummeted from 96.7% pre-war to a mere 40.7% [7]. This catastrophic drop is not due to patient negligence, but to a lethal combination of unavailable medications. Out of multiple centers, only four humanitarian centers, four public facilities, and one private pharmacy could meet the WHO standards of essential medication [10].

Among diabetic patients attending Gaza’s primary health centers during the conflict, 41% have reported missed insulin doses due to stockouts, 28% have experienced acute hyperglycemic episodes, and 9% have developed diabetic ketoacidosis (DKA) requiring emergency care [7]. These findings highlight the real and immediate clinical consequences of treatment interruption in a collapsing health system. The situation is perhaps most grim for the approximately 1,200 patients with end-stage renal disease, including 45 children, who are dependent on dialysis [3]. A health-facility-based survey found that only 38% of dialysis patients have been able to maintain their full treatment schedule during the conflict; over 60% have missed at least two sessions per week, and mortality reached 22% within three months of service interruption. Power outages and shortages of dialyzers and bicarbonate concentrate have forced most units to reduce treatment time from four to two hours, while 41% of patients have been treated with reused or improvised dialyzers [11]. With treatment centers destroyed or inaccessible, each missed session allows uremic toxins to accumulate, a condition that leads to multi-organ failure and death within weeks [12].

There are 350,000 patients in Gaza with chronic conditions, such as cancer and diabetes, in addition to 50,000 pregnant women who are struggling to access necessary care [13]. In a 2025 cross-sectional study of 512 pregnant women, it was reported that only 36.7% achieved the WHO-recommended eight antenatal visits, 28% delivered at home or in temporary shelters, and 15% of births were preterm. Low-birth-weight prevalence reached 10.8%, and maternal anemia exceeded 60% due to food insecurity and disrupted supplementation programs [14]. These data illustrate how prolonged siege conditions have dismantled essential chronic-care and reproductive-health services, transforming preventable conditions into life-threatening emergencies.

This slow-motion catastrophe is a textbook example of how conflict multiplies “indirect deaths.” It is feared that for every person killed by direct violence, as many as four others may die from the collapse of essential health services [15]. In Gaza, this secondary death toll is mounting daily, turning homes into hospices and manageable diagnoses into death sentences.

## The Collapse of Healthcare Systems and Supply Chains

The continuity of chronic disease care in Gaza is not just strained; it has been systematically dismantled by the destruction of the health system’s core components. When the conflict began in October 2023, the first weeks saw unprecedented bombardments that destroyed 18 hospitals, 32 primary care centers, and 11 water facilities, displacing over 1.4 million people and leaving more than 20,000 injured by the end of that year [16].

By mid-2024, the situation had deteriorated further. Gaza’s main tertiary hospital, Al-Shifa, became non-functional, while Al-Ahli Hospital remained the only major surgical facility, with three operating theaters serving over one million residents [17]. The escalating conflict and blockade caused severe shortages in essential medicines, electricity, and fuel, paralyzing intensive care, dialysis, and maternity services.

In June 2025, only 14 of Gaza’s 36 hospitals remained partially functional, operating under catastrophic conditions and overwhelmed by trauma cases [17, 18]. The WHO reported that Gaza City had only eight functioning operating rooms, while over 1,580 healthcare workers had been killed and hundreds detained [17]. A WHO assessment in September 2025 documented that 52% of essential medicines and 68% of medical supplies were completely out of stock [19].

This progressive collapse illustrates how the prolonged conflict has transformed Gaza’s already fragile healthcare network into a near-total vacuum of medical services. The continuing blockade, supply chain breakdown, and destruction of infrastructure have rendered routine chronic disease management virtually impossible, leaving the population reliant on sporadic humanitarian convoys and informal medical points.

## The Ceasefire as a Public Health Imperative

As the unrelenting assault on Gaza has precipitated a complete collapse of the health system, establishing a ceasefire is the single most critical public health intervention required to prevent further death and suffering. While the politics of the conflict are complex, the public health imperative is not; action is needed to prevent death, injury, and suffering from escalating. For public health professionals, a ceasefire is not a political concession but a fundamental necessity to address the unfolding humanitarian catastrophe [8].

Any medical or humanitarian solution remains futile under active bombardment, which severely obstructs access to basic medical services for civilians. An end to hostilities is the essential prerequisite to restoring a semblance of healthcare functionality. It is the only measure that can guarantee safe passage for humanitarian aid convoys and allow patients to reach the few remaining healthcare facilities. International humanitarian law requires that local arrangements, such as a suspension of fire, be made to permit the removal and transport of the wounded and sick.

Furthermore, a ceasefire is crucial for the protection of medical personnel, who are entitled to respect and protection under the Geneva Conventions. Without a ceasefire, these health workers face unacceptable risks, and the remaining health infrastructure is likely to be completely annihilated. Thus, a ceasefire is not merely a pause in killing but a necessary condition to uphold medical neutrality, deliver life-saving care, and begin the long process of rebuilding a decimated health system [20].

## Healthcare Recovery Cost in Post-Conflict Gaza

The destruction of Gaza’s healthcare system presents a multi-generational recovery challenge, with financial costs representing only a fraction of the total burden. Initial assessments from the World Bank and United Nations approximate the damage to public service infrastructure in the billions [21], while the World Health Organization estimates that a comprehensive reconstruction of the health sector will exceed $7 billion. This figure, however, does not account for the profound loss of human capital, including over 1,700 specialized medical personnel killed [22], or the complete collapse of the local economy, which has contracted by over 80% [23].

Given that the direct damages alone approach the entirety of the pre-conflict GDP of the Palestinian territories, a simple cost recovery timeline is inadequate. Realistically, reconstituting a functional healthcare system encompassing infrastructure, a trained workforce, and sustainable supply chains will necessitate a sustained international investment effort spanning several decades. This recovery is entirely contingent upon the establishment of lasting peace, stable governance, and unrestricted access for humanitarian and developmental aid.

Furthermore, the public health burden extends far beyond the direct and indirect mortality documented during the conflict. For the cohort of patients who survive this period of interrupted care, the long-term consequences will be severe and costly. Prolonged periods of uncontrolled hypertension will accelerate the onset of heart failure and stroke [5, 24], while sustained hyperglycemia in diabetic patients significantly increases the risk of irreversible complications such as retinopathy, neuropathy, and nephropathy [7, 9]. In Gaza, the breakdown of care continuity for NCD patients has already resulted in poor treatment adherence and critical medicine shortages [9]. This impending wave of complex, high-cost morbidity represents a profound, multi-generational strain on any future healthcare system and must be factored into recovery assessments alongside the rebuilding of physical infrastructure and the replacement of human capital. Rebuilding Gaza’s healthcare system will require decades and billions in investment. But before reconstruction, there must be stabilization: ensuring safe corridors for medication delivery, supporting local clinicians, and protecting what remains of the health infrastructure.

Furthermore, the long-term health consequences of this unprecedented conflict remain insufficiently understood. Establishing coordinated surveillance systems to document excess mortality, chronic disease progression, and emerging disabilities among Gaza’s population is essential to fully capture the hidden burden of the war. Ongoing WHO Public Health Situation Analysis (PHSA) reports and UN humanitarian health assessments should be expanded to include these indicators as part of post-conflict recovery planning. Continuous monitoring will not only quantify long-term impacts but also guide equitable rebuilding of the health system.

## Data Availability

All data and materials cited in this commentary are publicly available through the World Health Organization (WHO), United Nations (UN) agencies, and peer-reviewed journals as referenced in the text.

## Abbreviations

Abbreviation: Full Form
NCD: Non-communicable disease
WHO: World Health Organization
UN: United Nations
MoH: Ministry of Health
MP: Ministry of Health Primary Care Centre
DKA: Diabetic ketoacidosis
GDP: Gross domestic product
UNCTAD: United Nations Conference on Trade and Development
PHSA: Public Health Situation Analysis
